# Graphene Nanoplatelets’ Effect on the Crystallization, Glass Transition, and Nanomechanical Behavior of Poly(ethylene 2,5-furandicarboxylate) Nanocomposites

**DOI:** 10.3390/molecules27196653

**Published:** 2022-10-06

**Authors:** Dimitra Kourtidou, Maria-Eirini Grigora, Dimitrios Tzetzis, Dimitrios N. Bikiaris, Konstantinos Chrissafis

**Affiliations:** 1 Laboratory of Advanced Materials and Devices, School of Physics, Aristotle University of Thessaloniki, GR-541 24 Thessaloniki, Greece; 2 Digital Manufacturing and Materials Characterization Laboratory, School of Science and Technology, International Hellenic University, 14 km Thessaloniki—N. Moudania, GR-570 01 Thermi, Greece; 3 Laboratory of Polymer Chemistry and Technology, Department of Chemistry, Aristotle University of Thessaloniki, GR-541 24 Thessaloniki, Greece

**Keywords:** poly(ethylene 2,5-furandicarboxylate), nanocomposites, crystallization, nanomechanical properties

## Abstract

Poly(ethylene 2,5-furandicarboxylate) (PEF) nanocomposites reinforced with various content of graphene nanoplatelets (GNPs) were synthesized in situ in this work. PEF is a widely known biobased polyester with promising physical properties and is considered as the sustainable counterpart of PET. Despite its exceptional gas barrier and mechanical properties, PEF presents with a low crystallization rate. In this context, a small number of GNPs were incorporated into the material to facilitate the nucleation and overall crystallization of the matrix. Kinetic analysis of both the cold and melt crystallization processes of the prepared materials was achieved by means of differential scanning calorimetry (DSC). The prepared materials’ isothermal crystallization from the glass and melt states was studied using the Avrami and Hoffman–Lauritzen theories. The Dobreva method was applied for the non-isothermal DSC measurements to calculate the nucleation efficiency of the GNPs on the PEF matrix. Furthermore, Vyazovkin’s isoconversional method was employed to estimate the effective activation energy values of the amorphous materials’ glass transition. Finally, the nanomechanical properties of the amorphous and semicrystalline PEF materials were evaluated via nanoindentation measurements. It is shown that the GNPs facilitate the crystallization process through heterogeneous nucleation and, at the same time, improve the nanomechanical behavior of PEF, with the semicrystalline samples presenting with the larger enhancements.

## 1. Introduction

Petroleum-derived plastics have been used for more than 100 years in a wide range of applications due to their low cost, light weight, processability, and tailoring of their properties. However, their environmental impact comes at a great cost, Greenhouse gas emissions are high during their production and disposal, while the amount of oil-based waste produced increases rapidly [1]. For these reasons, over the past 40 years, there have been extensive efforts to develop new biobased materials produced by renewable energy, with a lower carbon footprint and competitive properties to rival their counterpart petroleum-based plastics. In this context, science and industry have turned their attention to carbohydrate vegetable and lignin feedstocks in order to develop new building blocks for the polymers’ production [2,3,4,5,6]. 

One of the most promising biobased monomers is 2,5-furandicarboxylic acid (2,5-FDCA), which belongs to the furan family and can be produced by the oxidative dehydration of glucose [7]. 2,5-FDCA is considered as a potential biobased replacement for terephthalic acid (TA), which is used for the production of a wide range of polyesters (PET, PPT, PBT) [7,8,9,10]. Poly(ethylene 2,5-furandicarboxylate) (PEF) is the most widely studied polyester derived from FDCA due to its excellent water permeability, gas barrier, and mechanical properties, and it presents with advantageous thermal properties compared to its counterpart, PET [7]. Many studies focusing on the analysis of the crystallization and structure of PEF have been published [11,12], since it is well known that the crystalline state of the polymer is inexplicably linked to its physical properties, e.g., its gas barrier and mechanical properties [13,14,15]. 

In-depth crystallization studies of PEF have revealed the difficulty of stimulating the crystallization of PEF due to its heavy furan ring and rigid chain backbone [15,16]. The crystallization of polymers can be promoted by incorporating a small number of nanofillers in the polymer matrix, facilitating the nucleation and the overall crystallization process [17,18,19,20]. Several PEF nanocomposites have been produced and studied in terms of their structure, crystallization, and thermal degradation [21,22,23,24,25]. Martino et al. prepared PEF nanocomposites using montmorillonite modified with organophilic ammonium cations (OMMT) as a nanofiller and showed that the melt crystallization rate slightly increased, while the crystallinity of the nanocomposite samples was greater compared to neat PEF [23]. Xie et al. also prepared PEF/OMMT nanocomposites by melt polycondensation in the presence of organic montmorillonite and studied their crystallization and mechanical properties, concluding that OMMT promoted the melt crystallization and improved the tensile modulus and strength of PEF [26]. Codou et al. synthesized PEF/nanocrystalline cellulose (NCC) via the melt extrusion procedure. It was shown that the PEF/NCC nanocomposites presented with twice the level of crystallinity of neat PEF, and the presence of NCC induced a higher crystallization rate with increasing filler content [27]. PEF nanocomposites with 2.5 wt. % multi-walled carbon nanotubes (MWCNTs), functionalized-MWCNTs, (carboxyl-MWCNTs and amino-MWCNTs), and graphene oxide (GO) were synthesized via melt polycondensation and studied in terms of their thermal degradation and crystallization by Lotti et al. The nanocomposites presented with faster crystallization rates and a higher nucleation density than neat PEF [21]. 

Since the crystallization of a polymer plays a key role in its final crystal structure and physical properties, the process of crystallization and its study are of great interest. Therefore, PEF nanocomposites using graphene nanoplatelets (GNPs) as a nanofiller in various loadings were prepared, and their isothermal and non-isothermal crystallization kinetics from the glass and melt states were investigated using the Avrami and Hoffman–Lauritzen theories. Initially, the GNPs’ dispersion in the PEF matrix was evaluated by employing scanning electron microscopy. The equilibrium melting temperatures of the prepared samples were calculated using Hoffman–Weeks plots. The effective activation energy of neat PEF and PEF/GNPs nanocomposites’ glass transition was also estimated based on the non-isothermal experimental data of the amorphous materials, using the Vyazovkin isoconversional method. Finally, the effects of the GNPs on the nanomechanical behavior of amorphous and semicrystalline PEF were studied via nanoindentation tests. To the best of our knowledge, PEF/GNPs nanocomposites have not yet been studied in terms of their crystallization and glass transition kinetics and their nanomechanical behavior. Incorporating a filler and investigating the final properties of the nanocomposite materials could lead to new insights and widen the range of their possible applications.

## 2. Results

Prior to the study of the crystallization of all the prepared materials, their molecular weights were calculated through intrinsic viscosity measurements and published in our previous work [24]. The reported M_n_ (g/mol) for the neat PEF, PEF/0.5 GNPs, PEF/1 GNPs, and PEF/2.5 GNPs are 12 × 10^3^, 7 × 10^3^, 11 × 10^3^, and 7 × 10^3^, respectively. 

### 2.1. SEM Observations of the PEF and PEF/GNPs Nanocomposites

**Figure 1:**
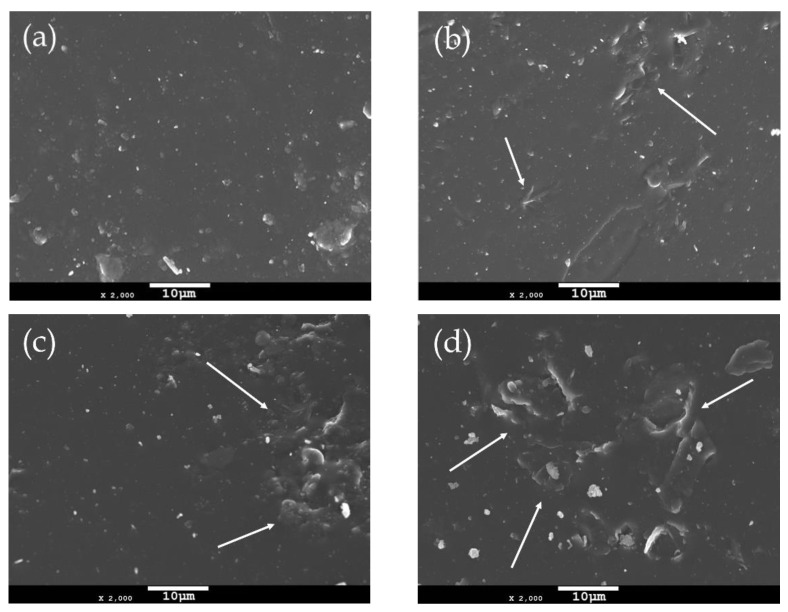
SEM images of the surfaces of (**a**) neat PEF and PEF/GNPs, nanocomposites containing (**b**) 0.5, (**c**) 1, and (**d**) 2.5 wt. % GNPs.

The incorporation of filler particles into a polymer matrix changes its morphology depending on the characteristics of the reinforcing filler. Thus, the surface morphology of neat PEF and PEF/GNP nanocomposites has been investigated using scanning electron microscopy (SEM). Figure 1 presents the surfaces of the annealed (at 418 K) PEF materials at ×2000 magnification. The neat PEF shown in Figure 1a presents with a smooth and uniform surface, absent of voids or cracks. On the other hand, the PEF/GNP nanocomposites present with a rather different surface morphology. Increasing the number of GNPs in the PEF matrix, it can be seen in Figure 1b–d that the roughness increases due to the presence of the near-surface GNPs. The number and the size of these surface inhomogeneities (examples are shown with arrows) increase with higher GNP loadings. The even larger size of these surface anomalies in the case of PEF/2.5 GNPs indicates the formation of the filler’s aggregates.

As seen from the SEM images of the PEF/GNP nanocomposites, no detached filler particles, cracks, or voids were found, indicating the successful incorporation of the filler in the PEF matrix. GNPs are characterized by hydrophobic features that make them compatible as fillers with the PEF matrix due to the polarity of its furan ring [28]. This high affinity between the GNPS and PEF results in the selected filler’s successful insertion into the polymer matrix, as confirmed by the SEM images, despite the formation of aggragates in the case of PEF/2.5 GNP nanocomposite.

### 2.2. Crystallization of the Neat PEF and PEF/GNP Nanocomposites

**Figure 2:**
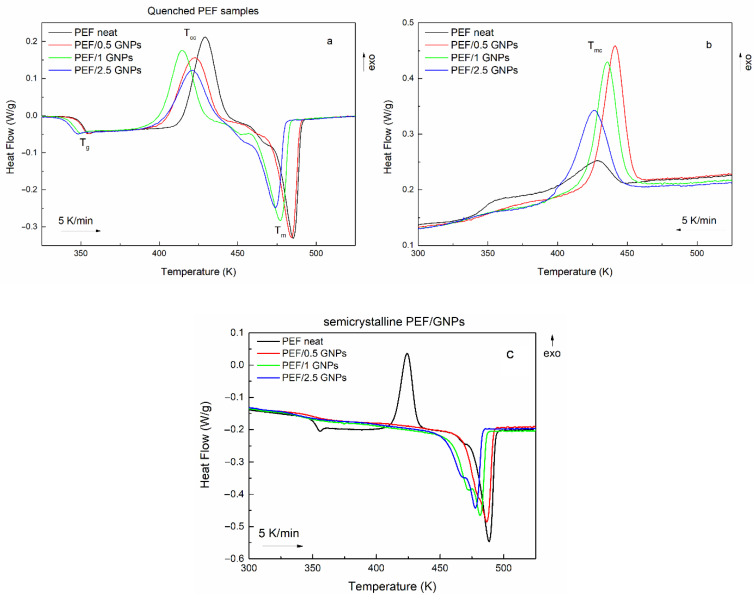
Heating curves of the quenched neat PEF and PEF/GNP nanocomposites (**a**), their subsequent cooling curves (**b**), and their heating curves after their melt crystallization (**c**) (the heating/cooling rate is 5 K/min).

The initial evaluation of the cold and melt crystallization behavior of the prepared materials was conducted through DSC heating scans of the quenched samples and their subsequent cooling at a 5 K/min rate. Figure 2 shows the heating and cooling curves of neat PEF and PEF/GNP nanocomposites. The glass transition (T_g_), cold crystallization (T_cc_), melting (T_m_), and melt crystallization (T_mc_) temperatures, as well as their corresponding enthalpies, are presented in Table 1. The degree of crystallinity of all the prepared materials was calculated using the melting enthalpies of the semicrystalline samples during the subsequent heating (Figure 2c) after the cooling scans at a 5 K/min rate, using Equation (1) [29]:

(1) $$X_{c}=\frac{{\Delta H}_{m2}-{\Delta H}_{\mathrm{cc}2}}{{\omega \Delta H}_{m}^{0}}\times 100\%$$

where ΔH_m2_ is the melting enthalpy, as presented in Table S1 in the Supplementary Materials report, ΔH_cc2_ (Table S1) is the cold crystallization enthalpy, ω is the weight fraction of the PEF, and ΔH_m_^0^ is the melting enthalpy of the 100% crystalline PEF (137 J/gr) [30].

It was observed that the incorporation of GNPs shifts the cold crystallization to lower temperatures, suggesting a nucleation effect. However, the PEF/2.5 GNPs do not follow this trend, meaning that their T_cc_ is higher than that of the PEF/1 GNPs. This can be attributed to the difficulty of the processes of the macromolecular chains’ diffusion and, subsequently, crystal formation during the process due to the dense dispersion of the platelets in the bulk polymer. The melting temperatures also decrease with increasing filler content, where the incorporation of 1 and 2.5 wt. % GNPs shifts the T_m_ to considerably lower temperatures. The lower melting temperatures and enthalpies of PEF/1 and 2.5 GNPs suggest the formation of less thermally stable crystals during the cold crystallization process. A shoulder peak in the initial stages of melting of all the prepared materials can be observed, indicating the recrystallization and melting of the macromolecular chains [11] between the cold crystallization and melting processes. 

The crystallization of all the prepared materials upon cooling at a 5 K/min rate is presented in Figure 2b. Neat PEF presents with a broad and weak exothermic peak, while the PEF/GNP nanocomposites’ crystallization is characterized by a more profound exothermic peak at higher temperatures than neat PEF, meaning that crystal formation is favored in the presence of a nucleating agent, such as GNPs. The nanocomposites’ crystallization peak broadens and shifts to lower temperatures with an increasing GNP content, suggesting a slower crystallization rate with high loadings of the filler.

The subsequent melting of the semicrystalline materials, as presented in Figure 2c, reveals that neat PEF’s melt crystallization is incomplete due to the lack of a nucleating agent, which leads to a distinct cold crystallization peak during the heating of semicrystalline PEF [11]. On the other hand, the semicrystalline PEF/GNP nanocomposites’ heating curves do not present a cold crystallization peak, suggesting that the crystalline formation occurs during cooling and is favored by the presence of GNPs in the bulk polymer. This is also shown by the crystallinity degree reached during the melt crystallization process of the prepared materials, where the PEF/GNP nanocomposites present with considerably higher crystallinity compared to neat PEF. The cold crystallization temperature of the neat semicrystalline PEF (Figure 2c) shifts to lower temperatures than the amorphous one due to the presence of crystals formed during cooling, acting as nucleating sites for the subsequent cold crystallization and thus favoring the occurrence of the process at lower temperatures. 

The semicrystalline PEF/GNP nanocomposites (Figure 2c) present with a profound double melting peak, which has been previously observed in the case of polymer nanocomposites [11,12,30,31,32]. Double melting peaks have been attributed to the melting recrystallization and remelting of the polymer chains, the presence of crystal modifications, such as two different crystal phases, the different lamellar thickness or crystal imperfections of the formed crystals, and species with different molecular weights. Considering that the XRD patterns of the semicrystalline prepared materials presented in our previous work [24] do not imply the presence of two different crystal phases, and the fact that the PEF/GNPs do not present with a cold crystallization peak, the double melting behavior is most probably connected to the formation of imperfect crystals with different lamellar thicknesses. These crystals have less thermal stability and melt at lower temperatures, inducing the earlier secondary melting peak [11,30].

**Figure 3:**
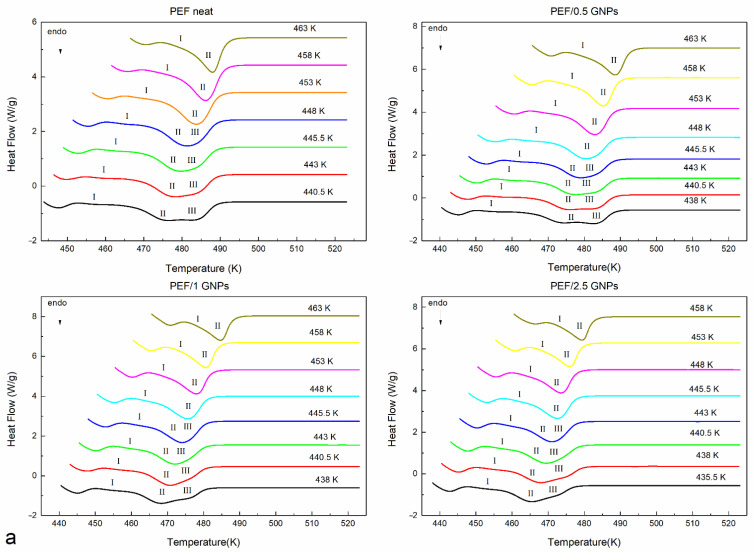

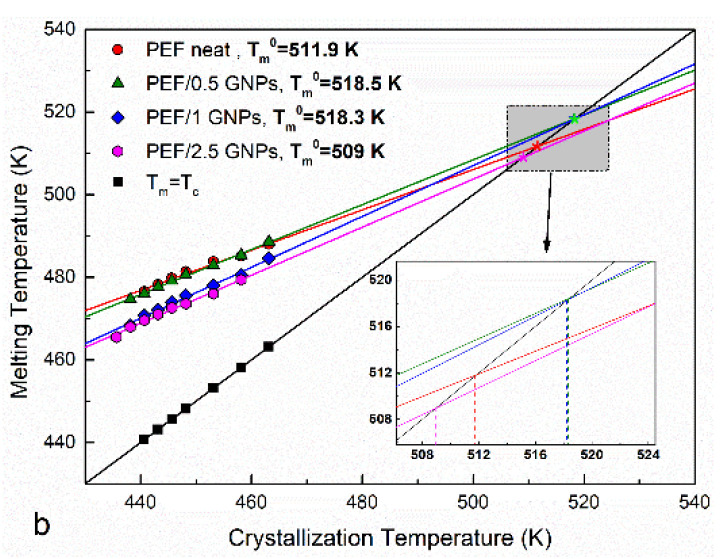
Subsequent melting curves after the isothermal melt crystallization at various temperatures (**a**) and Hoffman–Weeks plots (**b**) of the neat PEF and PEF/GNP nanocomposites.

The prepared materials’ equilibrium melting temperatures were also estimated using the Hoffman–Weeks method [33]. According to this method, all the prepared materials were initially crystallized from the melt isothermally at various temperatures. Their subsequent heating curves were recorded at a 15 K/min heating rate. The melting curves of the neat PEF and PEF/GNP nanocomposites after the isothermal crystallization are shown in Figure 3a. All the materials present with three melting peaks at crystallization temperatures below 448 K. It can be observed that the endothermic peaks I and III tend to merge with peak II as the crystallization temperatures increase, while for T_c_ above 448 K, peaks II and III finally merge. This melting behavior has been observed in the past [11,12,30,31], with most studies suggesting that, at lower temperatures, peak I occurs due to the melting of crystals formed in the early stages of secondary crystallization (formation of thinner lamella between the primary lamella). Peak II is attributed to the melting of the crystals formed upon primary crystallization, which are thermodynamically stable. The endothermic peak III is linked with crystals of a higher lamellar thickness formed during the heating of each material via the recrystallization process [3]. According to the Hoffman–Weeks theory [33], T_m_^0^ equals the crossing point of the extrapolation of the linear fit of the plot T_c_ − T_mII_ with the T_m_ = T_c_ line, as shown in Equation (2):

(2) $$T_{\mathrm{mII}}=T_{m}^{0}\left(1-\frac{1}{\beta }\right)+\frac{T_{c}}{\beta }$$

where T_mII_ is the melting temperature of the endothermic peak II (thermodynamically stable crystals formed at a temperature T_c_), and β is the thickening parameter, which is equal to L_c_/L_c_* and indicates the ratio of the mature crystallites’ thickness L_c_ to that of the initial ones L_c_*.

The Hoffman–Weeks plots of the recorded T_mII_, mapped against the corresponding isothermal crystallization temperatures of the neat PEF and PEF/GNP nanocomposites, are presented in Figure 3b. The calculated equilibrium melting temperatures are shown in the graph. As expected, the calculated T_m_^0^ values are between those reported by other authors for PEF (499–538 K) [11,12,30,31]. Generally, lower T_m_^0^ is a result of a lower molecular weight. However, the PEF/0.5 GNPs have a lower M_n_ value compared to neat PEF, but a higher T_m_^0,^ which suggests their higher degree of crystal perfection due to the presence of GNPs in the polymer [34]. This applies to the PEF/1 GNPs, whose M_n_ is close to that of PEF. However, this is not the case for the PEF/2.5 GNPs, which present with a lower T_m_^0^ value, meaning that the crystals formed during the isothermal melt crystallization process are less perfect. Alternatively, this could also be a result of the lower molecular weight.

#### 2.2.1. Isothermal Crystallization Kinetics of the Neat PEF and PEF/GNP Nanocomposites

**Figure 4:**
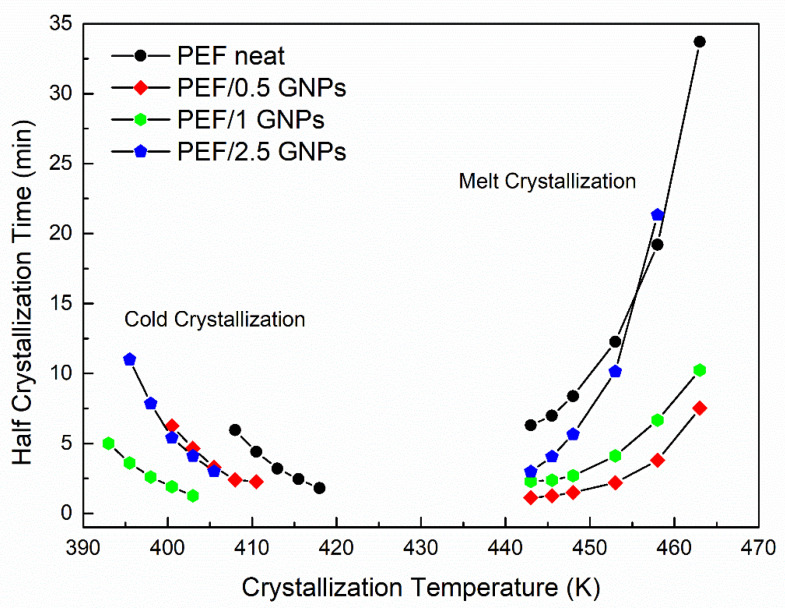
Half crystallization time vs. selected crystallization temperatures of the neat PEF and PEF/GNPs.

The GNPs’ effects on the cold and melt isothermal crystallization of PEF were examined in this work. The prepared materials were induced to isothermal crystallization from the glass at temperatures between 398 and 410.5 K, while the isothermal crystallization from the melt was conducted in a temperature range of 443–463 K, depending on the sample. The degree of conversion (or relative degree of crystallinity) of the crystallization processes α(t) was calculated using Equation (3):

(3) $$a\left(t\right)=\frac{\int_{0}^{t}\left(\frac{{\mathrm{dH}}_{c}}{\mathrm{dt}}\right)\mathrm{dt}}{\int_{0}^{\infty }(\frac{{\mathrm{dH}}_{c}}{\mathrm{dt}})\mathrm{dt}}$$

where dH_c_ represents the crystallization enthalpy at an infinitesimal time interval dt, and t and ∞ are the time passed during and at the end of the process, respectively. The half crystallization time (t_1/2_), i.e., the time the polymer needs to reach the 50% of the relative degree of crystallinity, for the crystallization from the glass and the melt was estimated. The values of t_1/2_ versus the crystallization temperature of the neat PEF and PEF/GNP nanocomposites are presented in Figure 4.

The PEF/GNP nanocomposites need less time to reach 50% crystallization compared to neat PEF, suggesting a clear nucleating effect even during the crystallization from the glass, where the process is controlled by diffusion rather than nucleation, which is the case for the melt crystallization of polymers [25,35]. More specifically, the t_1/2_ values of the PEF/GNPs are lower than that of neat PEF and decrease with an increasing filler content, except in the case of the PEF/2.5 GNPs. The molecular weight of the samples does not appear to play a primary role in this retardation, since lower molecular weights (and, therefore, shorter macromolecular chain lengths) should facilitate the diffusion of the polymer chains [36], accelerating the overall crystallization from the glass, which is not the case for the PEF/2.5 GNPs (M_n_ = 7 × 10^3^ g/mol). The filler’s dispersion and shape characteristics (aspect ratio, dimension), as well as its content, can significantly affect the overall crystallization from the glass state. The dense dispersion of GNPs in the case of the PEF/2.5 GNP nanocomposite and the formed aggregates, as seen in the SEM images, hinders the diffusion of the macromolecular chains, thus causing the retardation of the crystallization compared to the PEF/1 GNP nanocomposite. 

The half crystallization time of the PEF/GNPs, concerning the crystallization from the melt state, generally adopts lower values compared to neat PEF. While the nanocomposites crystallize faster than neat PEF within the widest range of temperatures, the t_1/2_ values increase with increasing filler content. This suggests that nucleating phenomena take place with a threshold effect. Increasing the GNP content resulted in larger aggregates, meaning a decrease in the specific surface area, i.e., lower surface areas available to induce the nucleation of the polymer chains. 

In order to gain better insight into the crystallization of the neat PEF and PEF/GNPs nanocomposites from the glass and melt states, isothermal crystallization kinetic analysis was carried out using the Avrami and Hoffman–Lauritzen (H-L) theories. These theories are commonly used to study crystallization kinetics and are based on different assumptions. 

The main assumption of the Avrami theory is that the nucleation rate of the polymer chains is constant, and the crystal growth is linear [37,38]. The evolution of the degree of conversion is mathematically described by Equation (4):

(4) $$\alpha \left(t\right)=1-e^{-{\mathrm{kt}}^{n}}$$

where n is the Avrami exponent, which provides information about the formed crystals’ dimensionality, and k is the rate constant that depends on the crystal growth and nucleation. Through linear fitting to the experimental data using the double logarithmic form of Equation (4), the values of n and k can be estimated:

(5) log(−ln[1−α(t)])=logk+nlogt

As mentioned before, the Avrami theory presents several limitations. It refers to single-step processes [25] and does not take into account the secondary nucleation phenomena which are common in polymer crystallization [25]. Thus, this theory is commonly used to study the crystallization process as a reasonable approximation [25]. Therefore, the Avrami theory was applied to the experimental data, and the values of the rate constant k and exponent n were calculated for both the isothermal cold and melt crystallization data of all the prepared materials. The plots of log(−ln [1-α(t)]) against logt for all the crystallization temperatures of the neat PEF and PEF/GNPs nanocomposite are presented in Figure S1. Accordingly, the resulting parameters n and logk are presented in Table S2. The half crystallization time was estimated by Equation (6) using the calculated k and n values:

(6) $$t_{1/2}={\left(\frac{\ln2}{k}\right)}^{1/n}$$

**Figure 5:**
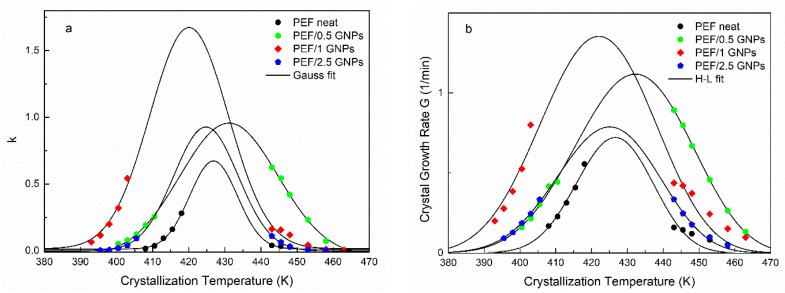
Crystallization rate constant k calculated by Avrami analysis together with the Gaussian fitting (**a**) and crystal growth rate, as well as the Hoffman–Lauritzen fittings for the isothermal melt and glass crystallization (**b**) vs. selected isothermal crystallization temperatures of the neat PEF and PEF/GNP nanocomposites.

The resulting t_1/2_ values were equal to those shown in Figure 4 for all the prepared materials. Although the n values are linked with the dimensionality of the formed crystals, as mentioned above, their interpretation could lead to misleading conclusions, considering the limitations of the Avrami model in regard to the polymers’ crystallization [25,39]. Nevertheless, by plotting the k values against the corresponding crystallization temperatures and applying a Gaussian fit, one can acquire information on each material’s crystallization rate and the maximum crystallization rate constant temperature. Figure 5a presents the resulting k values vs. the crystallization temperatures of the neat PEF and PEF/GNP nanocomposites along with the Gaussian fitting. During cold crystallization, the PEF/1 GNPs present with higher crystallization rate constants, while during the melt crystallization, the PEF/0.5 GNPs’ k values are higher compared to the rest of the nanocomposites and neat PEF. In any case, the crystallization rate constant k of the nanocomposites is larger than that of neat PEF. The maximum crystallization rate constant temperatures for the neat PEF, PEF/0.5 GNPs, PEF/1 GNPs, and PEF/2.5 GNPs are 428, 431, 421, and 425 K, respectively. 

Following the Avrami analysis, the isothermal crystallization of the prepared materials from the glass and melt states was also studied using the Hoffman–Lauritzen (H-L) secondary nucleation theory [40]. The linear growth rate of the crystallization process (G) can be estimated by using the relationship 1/t_1/2_ ≈ G, as shown by Chan et al. [41], assuming that the heterogeneous nuclei formation does not depend on the temperature and all the sites are activated at the same time [42]. According to H-L theory, the spherulitic growth rate can be estimated by Equation (7):

(7) $$G=G_{0}\exp\left[\frac{-U^{*}}{R\left(T_{c}-T_{\infty }\right)}]\exp[\frac{-K_{g}}{T_{c}\left(\Delta T\right)f}\right]$$

where G_0_ is the pre-exponential factor. The first exponential term expresses the diffusion process’ contribution to the growth rate, and the second term is correlated with the nucleation. The activation energy required for the transportation of a segment to the growing front is represented by U*, T_m_^0^ is the equilibrium melting temperature calculated above, ΔT = T_m_^0^ − T_c_ is the undercooling degree, f = 2T_c_/(T_m_^0^ − T_c_) is the correction factor, and K_g_ is the nucleation parameter. T_∞_ is a hypothetical temperature commonly set to 30 K below the glass transition temperature, when T < T_∞_ diffusion stops. K_g_ can be calculated for secondary or heterogenous nucleation using the following equation:

(8) $$K_{g}=\frac{{j\sigma \sigma }_{e}b_{0}T_{m}^{0}}{{\Delta h}_{f}k_{B}}$$

where j is relevant to the regime, j = 4 for regimes I and III, and j = 2 for regime II. The b_0_ is the thickness of a single stem on the crystal, σ is the lateral surface free energy, σ_e_ is the fold surface free energy, Δh_f_ is the enthalpy of fusion per unit volume of crystal, and k_B_ is Boltzmann’s constant [43]. By fitting Equation (7) to the experimental values of G for both the cold and melt crystallization, the parameters U* and K_g_ for the neat PEF and PEF/GNP nanocomposites can be estimated. Figure 5b presents the L-H fitting to the crystal growth rate G of all the prepared materials, and the resulting parameters are presented in Table 2. The U* values of all the samples are of the same order of magnitude as the universal value (6.3 kJ/mol [44]). The PEF/GNP nanocomposites present with a lower segmental activation energy, as expected. The PEF/1 GNPs have a lower U* value than the rest of the nanocomposites. This agrees with the initial observations, suggesting that the diffusion process is promoted in the cold crystallization region. Accordingly, the nucleation parameter K_g_ of the nanocomposite materials is lower than that of neat PEF and increases with increasing filler content. This indicates that heterogenous nucleation phenomena occur during the crystallization of the PEF/GNP nanocomposites from the glass and melt states. Since K_g_ is connected with the energy required to form critically sized nuclei, the resulting values suggest that lower numbers of GNPs facilitate the nucleation of the polymer chains and the initiation of the crystallization at lower or higher temperatures in the case of cold and melt crystallization, respectively. It was found by Berkel et al. that the molecular weight does not affect the values of U* and K_g_ [45]; thus, these differences between the neat PEF and PEF/GNP nanocomposites are solely due to the GNPs’ effect.

#### 2.2.2. Non-isothermal Crystallization of the Neat PEF and PEF/GNP Nanocomposites

The non-isothermal glass and melt crystallization of the prepared materials was also studied in this work. The heating curves of the amorphous materials and their subsequent cooling at heating/cooling rates of 1, 2.5, 5, 10, 15, and 20 K/min are presented in Figure S2 in the Supplementary Materials report. During the heating of the neat PEF and PEF/GNP nanocomposites, the cold crystallization peak shifts to higher temperatures with an increasing heating rate for all the prepared materials. During heating at slower heating rates, at a given temperature, the polymer chains have enough time to gain the energy required to diffuse and form crystals; thus, the cold crystallization process occurs at lower temperatures. Due to the overlapping of the cold crystallization peak with the melting peak at heating rates higher than 2.5 K/min, for all the prepared materials, the non-isothermal kinetic analysis was not conducted using model-free and model-fitting methods. When the cooling rate is higher than 10 K/min, neat PEF does not present with a melt crystallization peak, meaning that, at a specific temperature, the polymer chains do not have enough time to form crystals due to the lack of a nucleating agent. This is not the case for the PEF/GNP nanocomposites, which present with melt crystallization peaks at any cooling rate, indicating that the GNPs act as a heterogeneous surface, promoting the macromolecular chains’ nucleation and subsequent the formation of crystals.

**Figure 6:**
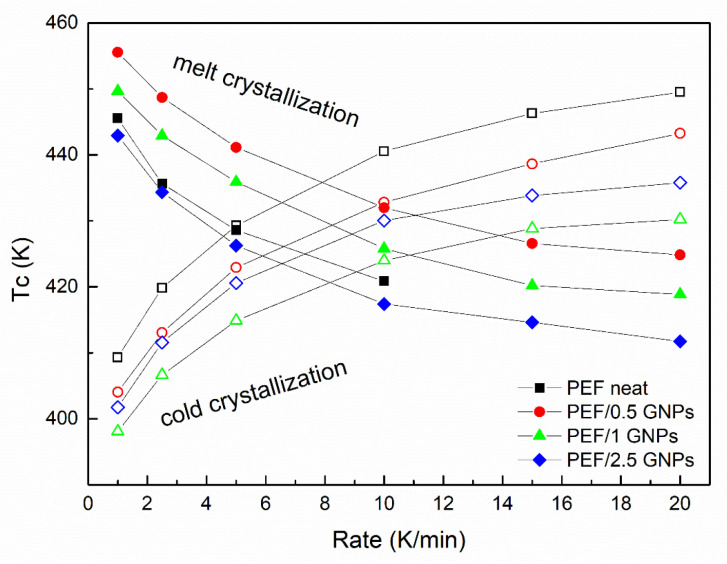
Cold and melt crystallization temperatures vs. heating/cooling rates of the neat PEF and PEF/GNP nanocomposites.

Figure 6 presents the cold and melt crystallization peaks vs. the heating/cooling rate of neat PEF and PEF/GNP nanocomposites. The cold crystallization peaks of the PEF/1 GNPs occur at lower temperatures at all the applied heating rates compared to neat PEF and the rest of the nanocomposites, while the melt crystallization peaks of the PEF/0.5 GNPs present at higher temperatures than the rest of the materials. These observations are in agreement with the isothermal observations (the cold crystallization of PEF/1 GNPs and melt crystallization of PEF/0.5 GNPs are faster compared to the rest of the prepared materials).

**Figure 7:**
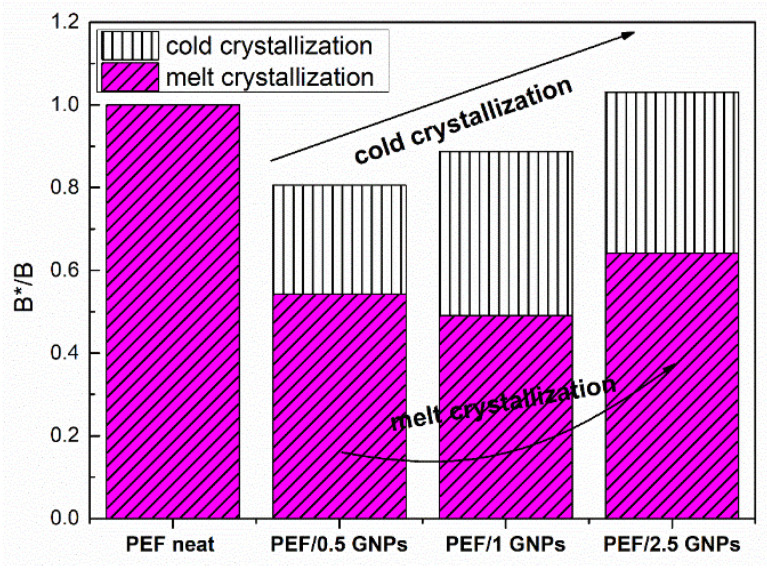
Nucleation activity of the neat PEF and PEF/GNPs nanocomposites, calculated by the Dobreva plots.

It is well known that the incorporation of a small number of nanofillers creates a nucleation effect during the crystallization of a polymer. This nucleation effect can be observed in the case of PEF/GNP nanocomposites in both the cold and melt crystallization of the materials, as previously mentioned. For this reason, the nucleation activity φ is also estimated using the non-isothermal crystallization data, applying the semi-empirical model of Dobreva and Gutzow [46,47]. This model suggests that when φ ≈ 0, the filler’s nucleation activity is extremely high, while when φ ≈ 1, the nucleation activity is considered absent. Following the Dobreva and Gutzow approach, φ is given by Equation (9):

(9) $$\varphi =\frac{B^{*}}{B}$$

where B is a parameter calculated experimentally by the slope of Equation (10), presented below, and is associated with the specific surface energy [46]:

(10) $$\ln\beta =\mathrm{Const}-\frac{B}{{\Delta Τ}_{p}^{2}}$$

where β is the heating/cooling rate, ΔΤ_p_ = Τ_cc_ − Τ_g_ for the cold crystallization data [48], and ΔΤ = Τ_m_ − T_mc_ for the melt crystallization data. B can be evaluated using the slopes of the plots lnβ against 1/(ΔΤ_p_)^2^. B is replaced by B* when a nucleating agent is incorporated into the polymer matrix. Figure 7 presents the resulting B*/B ratio of the neat PEF and PEF/GNPs for the cold and melt crystallization, while the Dobreva plots are presented in Figure S3 in the Supplementary Materials report.

As seen in Figure 7, the nucleation activity of the PEF/GNPs is more profound during the melt crystallization process, as expected, since the crystallization rate is controlled by nucleation phenomena, while the cold crystallization is controlled by diffusion. As seen above, the PEF/0.5 GNPs’ melt crystallization peaks are higher at any given cooling rate. However, the PEF/1 GNP nanocomposite presents a slightly greater nucleation activity. The B*/B ratio of the nanocomposites, in the case of the crystallization from the glass, increases with the increasing filler content. This can be attributed to the hindrance of the diffusion caused by the denser dispersion of GNPs in the polymer matrix.

#### 2.2.3. Glass Transition Kinetics of the Amorphous Neat PEF and PEF/GNP Nanocomposites

An analysis of the glass transition kinetics was also conducted in this work, using the heating DSC curves of the amorphous PEF and its nanocomposites. The curves at heating rates of 5, 10, 15, and 20 K/min were selected in order to examine the glass transition behavior of the prepared materials. The effective activation energy of the glass transition process was calculated using the advanced isoconversional method proposed by Vyazovkin [49,50]. According to this method, the effective activation energy is calculated at any specific value of the degree of conversion α for a series of n experiments executed under different temperature programs, T_i_ (t), by finding E_α_, which minimizes the function:

(11) $$\Phi \left(E_{\alpha }\right)=\sum_{i=1}^{n}\sum_{j=i}^{n}\frac{J\left[E_{\alpha },T_{i}\left(t_{\alpha }\right)\right]}{J\left[E_{\alpha },T_{j}\left(t_{\alpha }\right)\right]}$$

where

(12) $$J\left[E_{a},T_{i}\left(t_{a}\right)\right]=\int_{t_{a-\Delta \alpha }}^{t_{a}}e^{-\frac{E_{a}}{{\mathrm{RT}}_{i}\left(t\right)}}\mathrm{dt}$$

The subscript α represents the values at a given degree of conversion. The value of α varies between ∆α and 1–∆α, with a step of ∆α = m–1, m being the chosen integrals’ number. The minimization procedure is repeated for each value of α, indicating the activation energy’s dependence on the degree of conversion.

**Figure 8:**
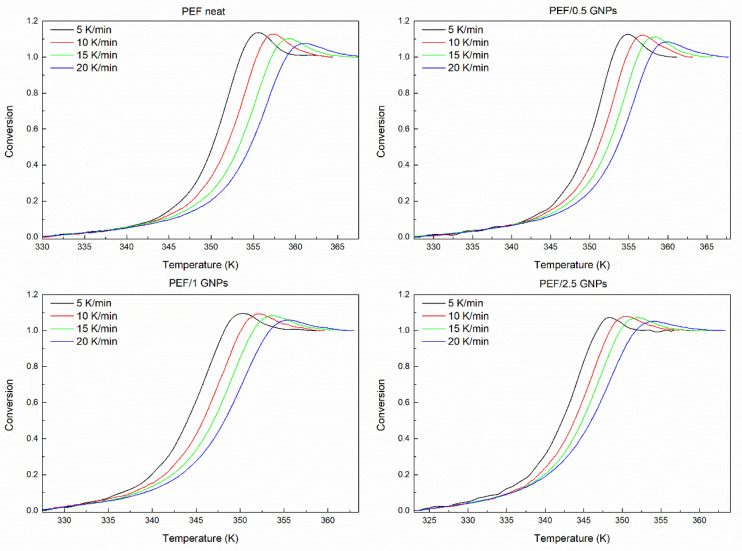
Conversion curves of the glass transition area of all the prepared materials.

The degree of conversion of the glass crystallization process is estimated as the normalized heat capacity determined by the DSC curves, using the following equation [51]:

(13) $$C_{p}^{N}=\frac{{\left.\left(C_{p}-C_{pg}\right)\right|}_{T}}{{\left.\left(C_{pe}-C_{pg}\right)\right|}_{T}}=a$$

where C_p_, C_pg_, and C_pe_ are the current, glassy, and equilibrium heat capacities, respectively. The glass transition’s degree of conversion of the prepared PEF materials is presented in Figure 8. The computation can be executed from C_p_^N^ = 0 to either 1 or the maximum C_p_^N^ value. The estimated E_α_ is not affected by the selected option of the limit [52]. 

**Figure 9:**
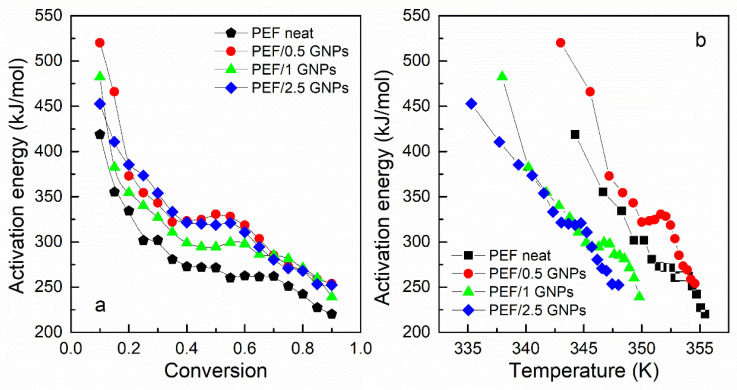
Activation energy of the glass transition vs. conversion (**a**) and temperature (**b**) of the neat PEF and PEF/GNP nanocomposites.

The glass transition’s effective activation energy for the prepared materials versus the degree of conversion and temperature is presented in Figure 9. All the materials’ E_α_ decreases rapidly with increasing temperature and the conversion from the glassy to the rubbery state. This is a common aspect of polymers’ glass transition activation energy and is attributed to the cooperative motion of the polymer chains during the heating of the sample [53]. The transition from the glassy to the rubbery state requires a large amount of energy and degree of cooperativity for the macromolecular chains to exceed the energy barrier in the early stages of the transition. The molecular motion increases and is facilitated by the increasing temperature, thus decreasing the effective activation energy.

As seen in Figure 9a, the E_α_ values of the PEF nanocomposites are slightly higher compared to that of neat PEF; however, the deviations are within the limits of the experimental error (15–25% [52]). The calculated values of E_α_, for neat PEF, are close to the ones estimated by Codou et al. [53]. Thus, it can be concluded that the glass transition is not greatly affected by the incorporation of the GNPs into the polymer matrix. However, the temperature dependence of E_α_, as presented in Figure 9b, demonstrates that the glass transition process of the PEF/1 GNPs and PEF/2.5 GNPs occurs earlier in the heating process, as observed in Table 1, where the T_g_ appears to shift to lower temperatures with the increasing filler content. This indicates that when the incorporation of the GNP content is larger than 0.5 wt. %, the overall glass transition is accelerated to obtain a lower T, confirming the findings of previous work [54]. The molecular weight of the samples was also considered during the analysis as a factor that plays a key role in the glass transition process, i.e., the lower molecular weight causes the shift of the glass crystallization to lower temperatures. However, the neat PEF and PEF/1 GNPs have similar M_n_, as do PEF/0.5 GNPs and PEF/2.5 GNPs, but their glass transition temperatures do not follow this behavior. Therefore, the glass transition temperature differences between the materials are most probably caused by the incorporation of the GNPs into the matrix. 

### 2.3. Nanoindentation Tests of the Neat PEF and PEF/GNP Nanocomposites 

**Figure 10:**
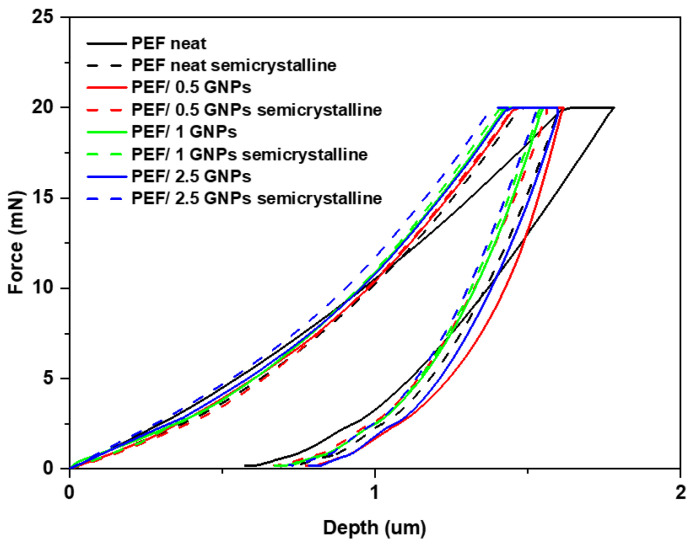
Comparison of the representative load–depth nanoindentation curves of the amorphous and semicrystalline neat PEF and PEF/GNP nanocomposites.

In this section, the reinforcing effect of the GNPs on PEF was investigated using nanoindentation testing. The representative load nanoindentation curves of both the amorphous and semicrystalline neat PEF and PEF/GNP nanocomposites are illustrated in Figure 10 as a function of depth. A higher degree of stiffness is observed as the load–depth curves shift towards the left with the increase in the GNPs in the matrix. For the PEF/GNP nanocomposites, the maximum nanoindentation depths at peak load varied between approximately 1.38 and 1.62 μm. The range of the nanoindentation depth ranged from 1.63 to 1.78 μm for the amorphous neat PEF and 1.47 to 1.60 μm for the semicrystalline neat PEF. For the amorphous PEF/GNP nanocomposites, the range of the nanoindentation depth ranged from 1.46 to 1.61 μm for the PEF/0.5 GNPs, 1.43 to 1.60 μm for the PEF/1 GNPs, and 1.42 to 1.55 μm for the PEF/2.5 GNPs. Additionally, in the case of the semicrystalline PEF/GNP nanocomposites, the range of the nanoindentation depth was 1.46 to 1.56 μm for the PEF/0.5 GNPs, 1.41 to 1.54 μm for the PEF/1 GNPs, and 1.39 to 1.53 μm for the PEF/2.5 GNPs.

**Figure 11:**
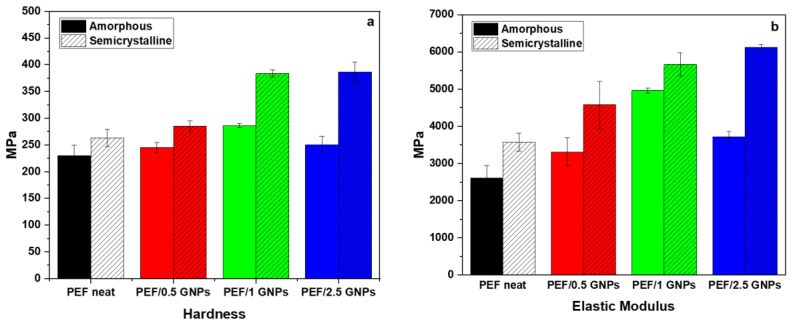
Comparison of (**a**) the hardness and (**b**) elastic modulus of the amorphous and semicrystalline neat PEF and PEF/GNP nanocomposites.

The average values of the hardness and elastic modulus of the amorphous and semicrystalline neat PEF and PEF/GNP nanocomposites are presented in Figure 11. According to the results, the semicrystalline PEF materials present a better nanomechanical behavior compared to their amorphous counterparts. The values of the hardness of the amorphous and semicrystalline neat PEF and PEF nanocomposites are reported in Table S3 in the Supplementary Materials report and Figure 11a. These results reveal that the presence of GNPs in both the amorphous and semicrystalline PEF/GNP nanocomposites enhances the hardness in comparison to the amorphous and semicrystalline neat PEF. Generally, the improvement in the nanomechanical properties, such as hardness, might be attributed to the strengthening of the dispersion, resulting from the addition of GNPs to the PEF matrix [55]. As shown in Figure 11a, the addition of 1 wt. % GNPs resulted in the highest hardness value of the amorphous PEF/GNPs, reaching a value of 286.3 MPa. On the contrary, the highest value of hardness, in the case of the semicrystalline samples, was achieved by the highest addition of 2.5 wt. % GNPs, reaching a value of 386.2 MPa. The higher crystal fraction, together with the presence of GNPs, might be the primary reason for the enhanced mechanical properties of the semicrystalline PEF/2.5 GNP nanocomposite. When compared to amorphous neat PEF, by adding 2.5 wt. % GNPs, the value of the hardness is increased by approximately 68%. 

The elastic modulus values of the amorphous and semicrystalline prepared materials derived from the nanoindentation tests are presented in Table S3 and Figure 11b. For the amorphous PEF/GNP nanocomposites, the elastic modulus increased significantly with the addition of 1% GNPs to the PEF matrix, reaching almost 4963 MPa, which is a 90% increase, as compared to the amorphous neat PEF. Furthermore, for the semicrystalline PEF/GNPs, the addition of 2.5 wt. %, reaching 6120 MPa, improved the value of elastic modulus by up to 135% in comparison to the amorphous neat PEF. Overall, the amorphous polymers were reported to show a lower elastic modulus than the semicrystalline ones, according to a prior study [56]. 

Generally, the semicrystalline materials presented here improved the hardness and elastic modulus compared to the corresponding amorphous materials, since the crystal content plays a key role in shaping a polymer’s mechanical properties. Highly ordered lamellae are more rigid and induce an enhanced hardness, strength, and elastic modulus due to more significant intermolecular bonding [57]. In the case of the amorphous samples, the GNPs’ incorporation resulted in an enhanced hardness and elastic modulus, which increased with the GNP content up to the value of 1 wt. % GNPs. The incorporation of 2.5 wt. % GNPs led to a somewhat lower hardness and elastic modulus values close to the ones of the PEF/0.5 GNPs. These values follow the trend of the molecular weights (as mentioned above), implying a possible relation between the M_n_ and their nanomechanical behavior [58]. However, this may also be attributed to the poorer dispersion state of the GNPs in the PEF/2.5 GNP nanocomposites, as seen from the SEM images. 

The semicrystalline samples, as already stated, present with higher hardness and elastic modulus values compared to the corresponding amorphous ones. The reinforcing effect of the GNPs on the semicrystalline matrix follows the trend of the filler content, i.e., the hardness and elastic modulus increase with increased GNP loading. This augmentation results from both the GNPs’ incorporation and the higher crystalline content induced by the filler, as mentioned earlier.

**Figure 12:**
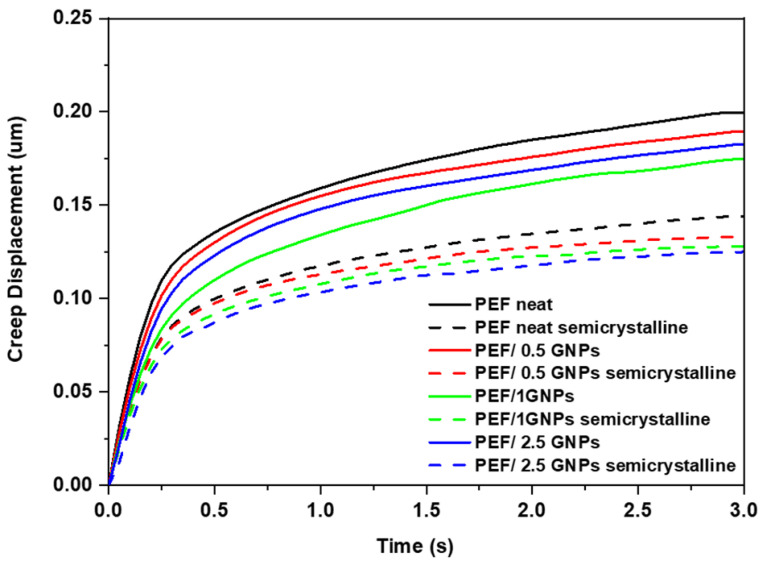
Comparison of representative creep displacement–time curves of the amorphous and semicrystalline neat PEF and PEF/GNP nanocomposites.

The creep displacement during the hold time was also calculated and is presented in Figure 12. It can be observed that the creep resistance of the GNP-reinforced nanocomposites increased with the increasing loading of the GNPs in the PEF matrix. The creep behavior of the neat PEF, as well as the PEF/GNP nanocomposites, differs between the amorphous and semicrystalline materials. Specifically, according to the nanoindentation creep displacement–time curves, the effect of annealing is clearly visible, and the creep displacement is greatly decreased for neat PEF and PEF/GNP nanocomposite specimens. The creep behavior improved with the increase in the GNP concentration in the PEF matrix, while the semicrystalline PEF/2.5 GNP nanocomposites exhibited the maximum creep resistance.

## 3. Materials and Methods

### 3.1. Materials 

Graphene nanoplatelets were incorporated into poly(ethylene 2,5-furandicarboxylate) (PEF/GNP nanocomposites). The GNPs, with the trade name M5, were supplied by XGSciences Inc (Lansing, Michigan, USA). They have an average diameter of 5 μm, an average thickness of approximately 6 to 8 nm, and a specific surface area of 120–150 m^2^ g. 

### 3.2. Synthesis of Poly(ethylene 2,5-furandicarboxylate)/Carbon Nanotube Nanocomposites (PEF/GNPs)

All the prepared materials were synthesized via a two-stage transesterification/polycondensation method in a glass batch reactor, as described in our previous work [24]. 

### 3.3. Characterization Techniques

#### Scanning Electron Microscopy (SEM) 

The surface morphology observations of all the prepared PEF samples were conducted via scanning electron microscopy (SEM). A Jeol JSM-7610F Plus scanning microscope was used, equipped with an AZTEC ENERGY ADVANCED X-act EDS Oxford analytical system, operating at 20 kV, with a probe current of 45 nA and counting time of 60 s (Jeol Ltd., Tokyo, Japan). All the samples were carbon-coated prior to the observations to ensure the good conductivity of the electron beam.

### 3.4. Differential Scanning Calorimetry (DSC)

Differential scanning calorimetry (DSC) was employed for the study of the neat PEF and PEF/GNP nanocomposites’ non-isothermal and isothermal crystallization using the Polyma 214 setup from NETZSCH, calibrated with indium and zinc standards. The mass of the selected samples was 10 ± 0.1 × 10 mg. 

In the non-isothermal crystallization analysis, all the samples’ thermal history was erased by heating from 273 K to 523 K at a heating rate of 20 K/min under a nitrogen flow of 60 mL/min. The samples were held at this temperature for 5 min. Then, to obtain the amorphous materials, the samples were cooled to 273 K at a heating rate of 100 K/min and were held at this temperature for 5 min. Afterward, for the cold crystallization kinetic analysis, the samples were heated at heating rates of 1, 2.5, 5, 10, 15, and 20 K/min for the amorphous materials. When the temperature reached 523 K, the samples were held at this temperature for 5 min. The non-isothermal glass transition analysis was performed at heating rates of 5, 10, 15, and 20 K/min, respectively. The activation energies of the prepared samples’ glass transition were calculated using the NETZSCH Kinetics Neo software (NETZSCH, Selb, Germany).

Isothermal experiments were also performed at temperatures ranging from 398 to 410.5 K, depending on the crystallization temperatures of each sample. To erase all the thermal history, each sample was initially melted at 523 K at a 20 K/min rate and held at that temperature for 5 min. Then, the samples were cooled at a 100 K/min rate at 273 K and subsequently heated rapidly at 70 K/min to the desired crystallization temperature and held at that temperature until the cold crystallization process’ completion. Afterward, the samples were heated at a 15 K/min heating rate to 523 K. For the isothermal melt crystallization analysis, the samples were initially heated at 523 K at a 20 K/min rate and held at that temperature for 5 min to erase all the thermal history. Each material was then cooled quickly at crystallization temperatures (T_mc_) varying between 443 and 463 K depending on the sample at a 70 K/min cooling rate down to the desired melt crystallization temperature and held to that T_mc_ until the completion of the crystallization process. Afterward, each sample was heated up to 523 K with a 15 K/min rate.

### 3.5. Nanoindentation Tests

The nanomechanical properties of the amorphous and semicrystalline neat PEF and PEF/GNP nanocomposite materials were studied by nanoindentation testing. The nanoindentation measurements, using a dynamic ultra-microhardness tester DUH-211S (Shimadzu Co., Kyoto, Japan), were conducted using a 100 nm-radius triangular pyramid indenter tip (Berkovich-type indenter) at room temperature (23 °C). The nanoindenter was loaded onto the surface of the films until a load peak of 20 mN was reached, and this was held for 3 s. Subsequently, the nanoindenter was unloaded, leading to the value of zero. The indentation depth was recorded as a function of load and the maximum load was applied to the nanoindenter during the creep time. In this study, the basic mechanical properties that were determined were the hardness and elastic modulus of the amorphous and semicrystalline neat PEF and their PEF/GNP nanocomposite samples using the Oliver and Pharr method and previous work [59,60,61,62,63,64,65]. The average value of ten measurements taken at different locations was used to calculate these properties. Moreover, the creep displacement was investigated during the nanoindentation testing. 

## 4. Conclusions

PEF/GNP nanocomposites were synthesized in situ in this work by the two-stage melt polycondensation method. The isothermal and non-isothermal crystallization processes of the prepared materials were studied using the DSC technique. The initial observations of the cold and melt crystallizations of both the amorphous and semicrystalline materials indicated that the incorporation of GNPs shifts the glass crystallization of the quenched materials to lower temperatures, suggesting a nucleation effect. However, PEF/2.5 GNPs do not follow this trend, possibly due to the difficulty of the process of the macromolecular chains’ diffusion, caused by the dense dispersion of the platelets in the bulk polymer. Crystallization measurements, upon cooling, revealed that crystal formation is favored in the presence of the nucleating agent; however, the nanocomposites’ crystallization rate lowers with increasing filler loadings. The Avrami and H-L theories were employed for the isothermal crystallization kinetic analysis. The results indicate that during the cold crystallization, the PEF/1 GNP nanocomposite presents with the highest crystallization rate constants, while during the melt crystallization, the PEF/0.5 GNPs’ crystallization rate is higher compared to the rest of the nanocomposites and neat PEF. Specifically, the U* and K_g_ values derived by the H-L analysis suggest that lower numbers of GNPs facilitate the nucleation of the polymer chains and the initiation of crystallization at lower or higher temperatures in the case of the cold and melt crystallization, respectively. The nucleation activity of the GNPs was also investigated using the Dobreva method. The results show that the nucleation efficiency of the GNPs is more profound during the melt crystallization process, as expected, since the crystallization rate is controlled by nucleation phenomena. The glass transition kinetic analysis was achieved using the isoconversional method of Vyazovkin. The results indicate that the glass transition process of the PEF/1 GNPs and PEF/2.5 GNPs occurs earlier in the heating process. The amorphous and semicrystalline neat PEF and PEF/GNP nanocomposites were also examined using nanoindentation tests. As a result, the presence of the GNPs in the PEF matrix enhanced both the hardness and elastic modulus and improved the nanomechanical behavior of the PEF/GNP nanocomposites. The semicrystalline PEF/GNP nanocomposites presented with better mechanical properties compared to the amorphous ones. This improvement can mainly be attributed to both the annealing process and homogeneous dispersion of the GNPs in the matrix. Moreover, for the semicrystalline PEF/GNP nanocomposites, the best nanomechanical behavior was achieved by increasing the GNP content up to 2.5 wt. %. On the contrary, in the case of the amorphous PEF/GNP nanocomposites, the best results were observed in the case of the 1% GNP loading, possibly due to the formation of GNP aggregates in the PEF/2.5 GNP nanocomposite, as indicated by the SEM observations. This suggests that, in the case of the amorphous PEF materials, the GNP dispersion is the main control factor, in contrast to the semicrystalline samples, where the fraction of the crystalline phase also plays a key role.

## Figures and Tables

**Table 1:** Glass transition, cold crystallization, melting temperatures, and enthalpies of the quenched neat PEF and PEF/GNP nanocomposites and their subsequent melt crystallization temperatures and enthalpies at a heating/cooling rate of 5 K/min.

	Heating of Quenched Samples					Melt Crystallization		
Sample	T_cc_ (K)	T_g_ (K)	ΔH_cc_ (J/gr)	T_m_ (K)	ΔH_m_ (J/gr)	T_mc_ (K)	ΔH_mc_ (J/gr)	X_c_ %
neat PEF	429	352	44.4	486	47.8	429	16.4	13
PEF/0.5 GNPs	423	351	45.6	484	50.9	441	50.2	36.1
PEF/1 GNPs	415	346	45.1	477	47.7	436	51.9	36.4
PEF/2.5 GNPs	421	344	39.7	474	42	426	45.6	35.2

**Table 2:** Values of Kg and U*, derived by the H-L fitting to the crystallization growth rate G of the neat PEF and PEF/GNP nanocomposites.

Sample	K_g_ (K^2^) × 10^5^	U* (kJ/mol)	T_max_ (K)	R^2^
neat PEF	7.4	16.9	428	0.962
PEF/0.5 GNPs	3.6	8.5	432	0.995
PEF/1 GNPs	4.8	8.2	422	0.908
PEF/2.5 GNPs	5	12.7	425	0.965

## Data Availability

The data presented in this study are available on request from the corresponding author.

## Supplementary Materials

The following supporting information can be downloaded at: https://www.mdpi.com/article/10.3390/molecules27196653/s1, Table S1. Cold crystallization and melting temperatures and enthalpies of semicrystalline neat PEF and PEF/GNP nanocomposites during their heating at 5 K/min; Figure S1. Avrami plots of neat PEF (a), PEF/0.5 GNPs (b), PEF/1 GNPs (c), and PEF/2.5 GNPs (d); Table S2. Avrami exponent n and logarithm of rate constant k values for the isothermal cold and melt crystallization region of neat PEF and PEF/GNP nanocomposites; Figure S2: Heat flow curves during the heating of the quenched neat PEF and PEF/GNP nanocomposites (a) and their subsequent cooling (b) at rates of 1, 2.5, 5, 10, 15, and 20 K/min; Figure S3. Dobreva plots of neat PEF and PEF/GNP nanocomposites for the cold crystallization (a) and melt crystallization (b) processes; Table S3. Hardness and elastic modulus of the prepared amorphous and semicrystalline PEF materials.
