# Supplementary material for: Graphene Nanoplatelets’ Effect on the Crystallization, Glass Transition, and Nanomechanical Behavior of Poly(ethylene 2,5-furandicarboxylate) Nanocomposites
Source: Molecules. 2022 Oct 6;27(19):6653. doi: 10.3390/molecules27196653 (PMC9571983; doi:10.3390/molecules27196653)
Supplement: Supplementary file 1 [file molecules-27-06653-s001.zip › molecules-1909722-supplementary.pdf]

molecules

MDPI

Supplementary Material Report

Graphene Nanoplatelets' Effect on the Crystallization, Glass Transition, and Nanomechanical Behavior of Poly(ethylene 2,5-furandicarboxylate) Nanocomposites

Dimitra Kourtidou [1], Maria-Eirini Grigora [2], Dimitrios Tzetzis [2], Dimitrios N. Bikiaris [3,*] and Konstantinos Chrissafis [1,*]

[1] Laboratory of Advanced Materials and Devices, School of Physics, Aristotle University of Thessaloniki, GR-541 24 Thessaloniki, Greece

[2] Digital Manufacturing and Materials Characterization Laboratory, School of Science and Technology, International Hellenic University, 14 km Thessaloniki—N. Moudania, GR57001 Thermi, Greece

[3] Laboratory of Polymer Chemistry and Technology, Department of Chemistry, Aristotle University of Thessaloniki, GR-541 24 Thessaloniki, Greece

* Correspondence: dbic@chem.auth.gr

Table S1. Cold crystallization and melting temperatures and enthalpies of semicrystalline neat PEF and PEF/GNPs nanocomposites, during their heating with 5 K/min.

	Heating after cooling with 5 K/min			
**Sample**	T_{cc2} (K)	ΔH_{cc2} (J/gr)	T_{m2} (K)	ΔH_{m2} (J/gr)
**neat PEF**	424	33	488	50.9
**PEF/0.5 GNPs**	-	-	487	49.27
**PEF/1 GNPs**	-	-	482	49.3
**PEF/2.5 GNPs**	-	-	478	47

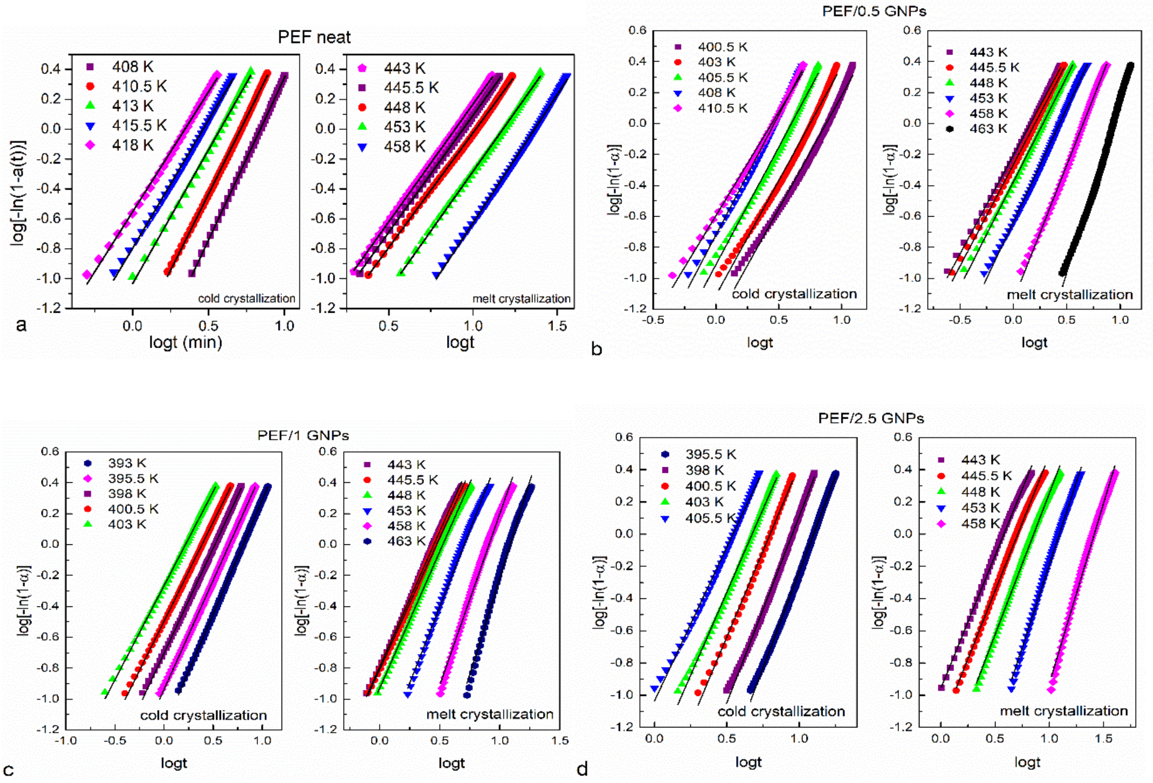

Figure S1. Avrami plots of neat PEF (a), PEF/0.5 GNPs (b), PEF/1 GNPs (c) and PEF/2.5 GNPs (d).

Table S2. Avrami exponent n and logarithm of rate constant k values for the isothermal cold and melt crystallization region of neat PEF and PEF/GNPs nanocomposites.

PEF neat			PEF/0.5 GNPs			PEF/1 GNPs			PEF/2.5 GNPs		
**Cold crystallization region**											
**T (K)**	**n**	**logk**	**T (K)**	**n**	**logk**	**T (K)**	**n**	**logk**	**T (K)**	**n**	**logk**
408	2.2	-1.8	400.5	1.5	-1.3	393	1.5	-1.2	395.5	2.4	-2.6
410.5	2	-1.5	403	1.5	-1.1	395.5	1.4	-0.9	398	2.3	-2.2
413	1.8	-1	405.5	1.5	-0.9	398	1.4	-0.7	400.5	2.1	-1.7
415.5	1.7	-0.8	408	1.6	-0.7	400.5	1.3	-0.5	403	2.1	-1.4
418	1.6	-0.6	410.5	1.3	-0.6	403	1.2	-0.3	405.5	1.9	-1
**Melt crystallization region**											
**T (K)**	**n**	**logk**	**T (K)**	**n**	**logk**	**T (K)**	**n**	**logk**	**T (K)**	**n**	**logk**
443	1.6	-1.4	443	1.3	-0.2	443	1.7	-0.8	443	1.6	-1
445.5	1.6	-1.5	445.5	1.3	-0.3	445.5	1.7	-0.8	445.5	1.7	-1.2
448	1.5	-1.6	448	1.4	-0.4	448	1.8	-0.9	448	1.8	-1.5
453	1.6	-1.9	453	1.5	-0.6	453	2	-1.4	453	2.1	-2.2
458	1.7	-2.3	458	1.7	-1.1	458	2.2	-2	458	2.2	-3.2
			463	2.2	-2.1	463	2.5	-2.7			

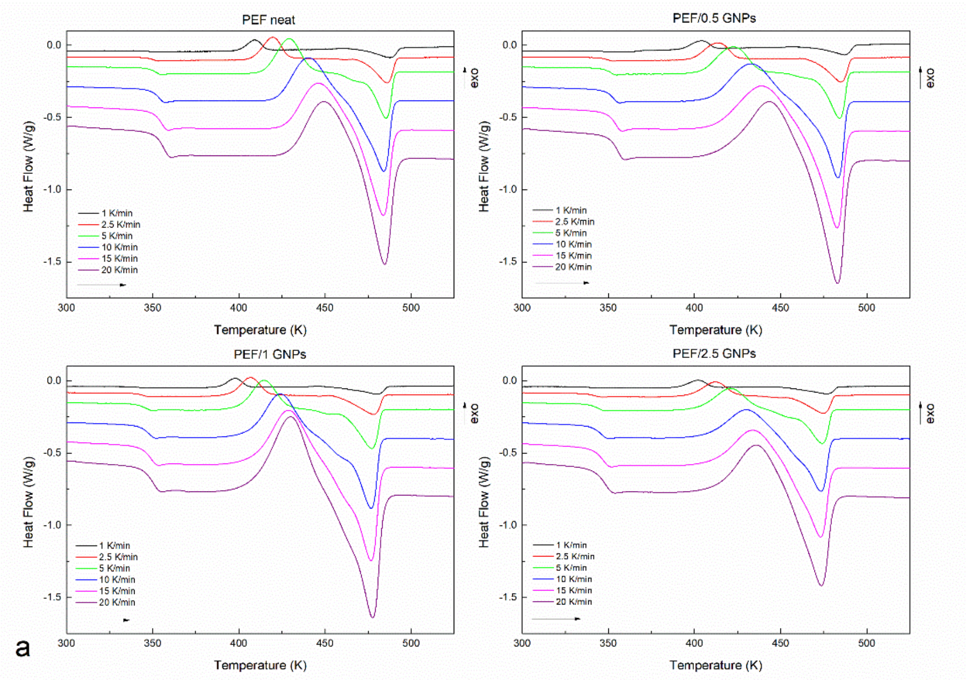

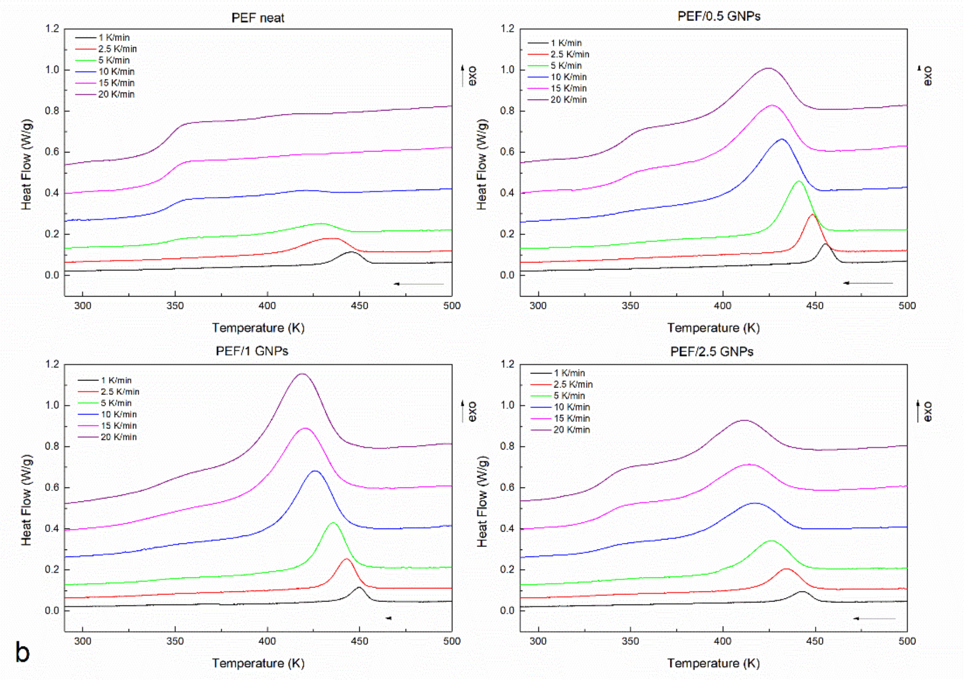

Figure S2. Heat flow curves during the heating of the quenched neat PEF and PEF/GNPs nanocomposites (a) and their subsequent cooling (b) with rates of 1, 2.5, 5, 10, 15, and 20 K/min.

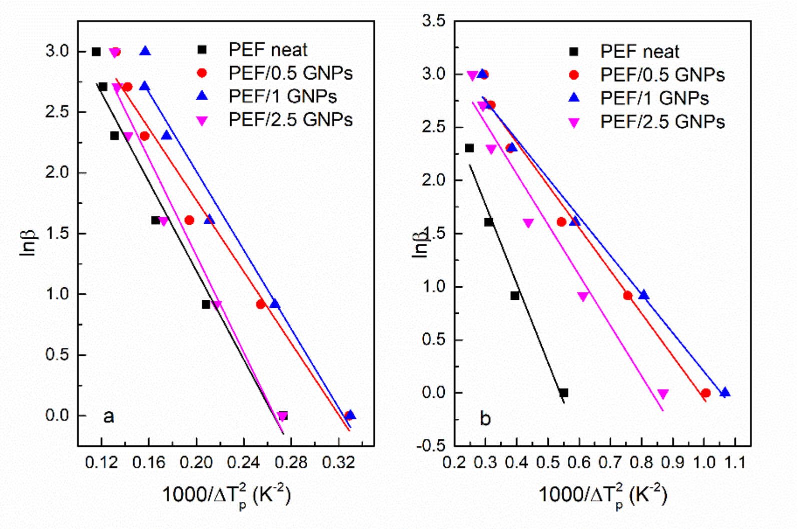

Figure S3. Dobreva plots of neat PEF and PEF/GNPs nanocomposites for the cold crystallization (a) and melt crystallization (b) processes.

Table S3. Hardness and Elastic modulus of the Amorphous and Semicrystalline prepared PEF materials.

	**Hardness**		**Elastic Modulus**	
	**Amorphous**	**Semicrystalline**	**Amorphous**	**Semicrystalline**
**PEF**	229.87 ± 19.84	263.07 ± 16.04	2606.30 ± 336.39	3572.50 ± 237.99
**PEF/0.5 GNPSs**	244.87 ± 9.29	284.87 ± 10.23	3312.75 ± 378.25	4578.00 ± 628.70
**PEF/1 GNPs**	286.34 ± 4.31	383.43 ± 6.69	4962.67 ± 69.10	5662.50 ± 315.26
**PEF/2.5 GNPs**	250.12 ± 16.03	386.15 ± 11.76	3720.67 ± 144.95	6120.00 ± 83.12
